# Weipiling decoction alleviates *N*-methyl-*N*-nitro-*N*′-nitrosoguanidine-induced gastric precancerous lesions via NF-κB signalling pathway inhibition

**DOI:** 10.1186/s13020-022-00663-y

**Published:** 2022-09-09

**Authors:** Penghui Yang, Hongmei Yang, Hengli Zhou, Qiuyue Li, Sufen Wei, Qi Wang, Yan Yan, Yongqiang Liu, Huafeng Pan, Siyi Li

**Affiliations:** 1 Science and Technology Innovation Center, Guangzhou University of Chinese Medicine, Guangzhou, 510405 China; 2 Department of Emergency Ward, The First Affiliated Hospital of Guizhou University of Chinese Medicine, Guiyang, 550001 China; 3 Department of Gastroenterology, The Second Affiliated Hospital of Guangzhou University of Chinese Medicine, Guangzhou, 510405 China; 4 Guangzhou University of Chinese Medicine, Guangzhou, 510405 China; 5 Research Center of Chinese Herbal Resources Science and Engineering, School of Pharmaceutical Sciences, Guangzhou University of Chinese Medicine, Guangzhou, 510405 China; 6 Joint Laboratory for Translational Cancer Research of Chinese Medicine of the Ministry of Education of the People’s Republic of China, Guangzhou, 510405 China; 7 International Institute for Translational Chinese Medicine, Guangzhou University of Chinese Medicine, Guangzhou, 510405 China; 8 Dongguan Institute of Guangzhou University of Chinese Medicine, Dongguan, 523808 China

**Keywords:** Weipiling decoction, Gastric precancerous lesion, NF-κB, *N*-methyl-*N*-nitro-*N*-nitrosoguanidine, Inflammation

## Abstract

**Aim of the study:**

We aimed to explore how weipiling (WPL) decoction WPL alleviates gastric precancerous lesions (GPLs) and uncover its anti-inflammatory roles in GPL treatment.

**Materials and methods:**

The anti-GPL action mechanisms of WPL were analysed using a network pharmacological method. The WPL extract was prepared in a traditional way and evaluated for its major components using high-performance liquid chromatography with tandem mass spectrometry (HPLC–MS/MS). BALB/c mice were exposed to *N*-methyl-*N*-nitro-*N*-nitrosoguanidine (MNNG) (150 μg/mL) for 6 weeks to induce GPLs. GPL mice were administered WPL (3.75 g/kg/day and 15 g/kg/day) for an additional 8 weeks*.* Haematoxylin and eosin (H&E) staining was used to investigate histological alterations in gastric tissues. Expression of the T helper 1 (Th1) cell markers CD4^+^ and interferon-gamma (INF-γ) were tested using immunohistochemistry (IHC). Inflammatory protein and mRNA levels in the nuclear factor kappa B (NF-κB) pathway were detected using western blotting and a quantitative reverse transcription polymerase chain reaction (RT-qPCR), respectively.

**Results:**

We identified and selected 110 active compounds and 146 targets from public databases and references. Four representative components of WPL were established and quantified by HPLC–MS/MS analysis. WPL attenuated MNNG-induced GPLs, including epithelial shedding, cavity fusion, basement membranes with asymmetrical thickness, intestinal metaplasia, dysplasia, pro-inflammatory Th1-cell infiltration, and INF-γ production, indicating that WPL prevents inflammation in the gastric mucosa. Furthermore, WPL reversed MNNG-induced activation of the IκB/NF-κB signalling pathway and subsequently attenuated the upregulation of inducible nitric oxide synthase (iNOS), cyclooxygenase-2 (COX-2), and nicotinamide adenine dinucleotide phosphate oxidase (NADPH oxidase (NOX)) family members NOX2 and NOX4.

**Conclusion:**

WPL attenuated GPLs by controlling the generation of pro-inflammatory elements and inhibiting the NF-κB signalling pathway in vivo.

## Introduction

Gastric cancer (GC) is a prevalent and fatal global malignancy [1, 2], and its incidence and mortality rates are still high in China [3]. Reportedly, approximately 26,380 new GC cases and 11,090 GC deaths in the U.S. in 2022 [4]. Surgery and chemotherapy are the main GC treatment strategies. However, most patients with GC benefit poorly from current treatments because they are usually diagnosed at advanced stages [5]. Therefore, early diagnosis and treatment are recognised as effective strategies to reduce GC mortality. To date, gastric precancerous lesions (GPLs), including chronic atrophic gastritis (CAG), dysplasia, and intestinal metaplasia (IM), are progressive stages prior to tumorigenesis in the gastric mucosa [6]. Moreover, it can prevent and reverse GPL progression via rational early treatment [7, 8]. Therefore, diagnostic and therapeutic management of GPLs is a critical strategy to reduce GC mortality.

GPLs are recognized as a chronic inflammatory state of the gastric mucosa. GPLs endure malignant transformation through long-term exposure to pro-inflammatory stimuli [9, 10], such as dietary patterns, obesity, smoking, and chronic infections [11]. One of the most common GPL stimuli is *N*-nitroso compounds (NOCs), which are frequently found in salted vegetables, fish, meat, and other salted foods [12]. NOCs chronically induce gastric carcinogenesis by promoting a pro-inflammatory response and stimulating DNA damage in the gastric mucosa [13, 14]. Therefore, *N*-methyl-*N*-nitro-*N*-nitrosoguanidine (MNNG), a carcinogenic NOC, is widely used to mimic GPLs in mice because of its DNA-damaging properties [11, 15–17] and inflammation-induced metaplasia in acid-secreting parietal cells [18]. Thus, MNNG-induced GPLs are a rational model to investigate the pathogenesis of gastric mucosa GPLs and inflammation.

Weipiling (WPL) decoction, a derivative of the Sijunzi decoction formula in ancient Chinese medicine, has been used to treat gastric disease and prevent GC for centuries in China. One significant symptom of GPL patients is summarised as “Qi deficiency” according to Chinese medicine theory and clinical practice. The WPL formula is frequently used to treat GPLs and prevent GC transfer due to its “Qi-refuelling” property. To date, WPL is still commonly prescribed to GPL patients in Chinese medicine hospitals [19], but its GPL-alleviating mechanism remains elusive. The composition of WPL, including *Astragalus mongholicus Bunge* (Huang Qi), *Atractylodes lancea (Thunb.) DC* (Bai Zhu), *Poria cocos (Schw.) wolf* (Fu Ling), *Pseudostellaria heterophylla (Miq.) Pax* (Tai Zi Shen), *Panax notoginseng (Burkill) F. H. Chen* (San Qi), *Scleromitrion diffusum (Willd.) R. J. Wang* (Bai Hua She Cao), *Curcuma zedoaria (Christm.*)*, Roscoe* (E zhu)*, Hericium erinaceus (Bull.),* and *Pers* (Hou Gu Jun) (Table 1), exerts potential GPL-alleviating and nuclear factor kappa B (NF-κB)-related anti-inflammatory activities. *Hericium erinaceus (Bull.) Pers* is critical in treating inflammatory diseases. Compounds extracted from *Atractylodes lancea (Thunb.) DC,* such as beta-eucalyptol and atractylone, inhibit inflammation by blocking the NF-κB signalling pathway [20–22]. A previous study also found that the main components of WPL, *Scleromitrion diffusum (Willd.) R. J. Wang* and *Hericium erinaceus (Bull.) Pers,* are used to treat gastrointestinal diseases, such as GC and CAG by attenuating tumour angiogenesis and proliferation and inducing apoptosis [23–31]. The major chemical components of WPL, including notoginsenoside R1, calycosin-7-*O*-beta-d-glucoside, astragaloside A, and adenine-9-β-d-ribofuranoside, were determined using high-performance liquid chromatography with tandem mass spectrometry (HPLC–MS/MS). However, the anti-inflammatory potential of WPL in GPLs remains unclear.

**Table 1:** Components and ratio of WPL

Local name	English name	Used part	Percentage (%)
Huang Qi	*Astragalus mongholicus Bunge*	Root	20
Bai Zhu	*Atractylodes lancea (Thunb.) DC*	Root	10
E Zhu	*Curcuma zedoaria (Christm.), Roscoe*	Root	9
Fu Ling	*Poria cocos (Schw.) wolf*	Sclerotium	10
Tai Zi Shen	*Pseudostellaria heterophylla (Miq.) Pax*	Root	10
San Qi	*Panax notoginseng (Burkill) F. H. Chen*	Root	5
Bai Hua She She Cao	*Scleromitrion diffusum (Willd.) R. J. Wang*	Grass	15
Hou Gu Jun	*Hericium erinaceus (Bull.) Pers*	Sclerotium	16
Shou Gong	*Gecko*	Total	5

Network pharmacology approaches, including cheminformatics, bioinformatics, network biology and pharmacology, are effective methods to study and elucidate the mechanisms of drug action [32]. In this research, we used network pharmacological analysis to illustrate the action mechanisms of WPL improving GPLs. Molecular docking was used to verify the binding ability of active compounds and potential targets in WPL. We constructed an MNNG-induced mouse model and attested the anti-inflammatory effect of WPL in in vivo experiments.

## Materials and methods

### Collecting WPL active ingredients and targets

The active ingredients were obtained from the Traditional Chinese Medicine Integrated Database (TCMID, http://www.megabionet.org/tcmid/) [33] and Traditional Chinese Medicine Systems Pharmacology Database and Analysis Platform (TCMSP, http://sm.nwsuaf.edu.cn/lsp/tcmsp.php) [34]. However, the active ingredients of *Hericium erinaceus (Bull.) Pers* and *Gecko* were not found in the TCMID or TCMSP. WPL-related targets were retrieved from TCMID, TCMSP, and the Search Tool for Interacting Chemicals (STITCH, http://stitch.embl.de) [35], then screened with a medium compound-target association score greater than 400 in the STITCH database. The target information was standardised using NCBI (https://www.ncbi.nlm.nih.gov/). Next, a drug-like analysis of WPL active components was carried out according to the quantitative estimate of drug-likeness (QED) proposed by Bickerton [36] with a QED ≥ 0.3. Subsequently, WPL active compounds and targets were screened using a binomial statistical model [37].

We searched for *Hericium erinaceus (Bull.) Pers* active components and targets in Herb Ingredients’ Targets (HIT, http://lifecenter.sgst.cn/hit/) and deleted some active constituents with no targets [38]. Additionally, calycosin 7-*O*-glucoside and notoginsenoside R1 targets, which have high drug activities, were retrieved from the PharmMapper (http://www.lilab-ecust.cn/pharmmapper) platform with a median Norm Fit ≥ 0.6008 and 0.4921, respectively, followed by further searches with a median Norm Fit ≥ 0.72915 and 0.586, respectively.

### Collecting GPL targets

GPL targets were collected from the GeneCards (https://www.genecards.org) database with “gastric precancerous lesions” as the search words, then screened with relevance scores ≥ 5.

### Protein–protein interaction (PPI) network construction

The PPI network was constructed using the STRING database (https://cn.string-db.org/) using common targets obtained from GPL cure targets and WPL-related targets using the Venny 2.1 tool. The PPI network was visualised using Cytoscape software 3.8.0.

### Function enrichment analysis

Gene Ontology (GO) and Kyoto Encyclopaedia of Genes and Genomes (KEGG) pathway enrichment analyses were performed using the clusterProfiler package 3.15.4. A significant enrichment analysis was set at *P* ≤ 0.01.

### Molecular docking analysis

Zinc (http://zinc15.docking.org) [39] and the Protein Data Bank (PDB, http://www.rcsb.org/pdb/) [40] were used to download the chemical structure of compounds and the three-dimensional structure of targets, respectively. Molecular docking analysis was performed using AutoDock Vina 1.1.2 [41] and plotted using PyMol 2.3.0 [42].

### Reagents and antibodies

Methanol, acetonitrile, and HPLC-grade acetic acid were purchased from Merck (Darmstadt, Germany). The reference substances *notoginsenoside R1*, *calycosin-7-O-beta-d-glucoside*, *astragaloside A,* and *adenine-9-β-d-ribofuranoside,* with HPLC-verified purities > 98%, and vitamin B12 (VitB12) were obtained from Meilun Biotechnology Co., Ltd. (Dalian, China). MNNG was provided by the Tokyo Chemical Industry (TCI) (Tokyo, Japan). Primary antibodies against inducible nitric oxide synthase (iNOS, Abcam, Cambridge, MA, USA, ab178945), cyclooxygenase-2 (COX-2, Abcam, ab179800), NOX-2 (Abcam, ab129068), NOX-4 (Abcam, ab154244), NF-κB p65 (8242S, Cell Signalling Technology (CST), Beverly, MA, USA), phospho-NF-κB p65 (CST, 3033S), IκB-α (CST, 4814S), phospho-IκB-α (CST, 2859S), CD4^+^ (CST, 93518S), interferon-gamma (IFN-γ, CST, 8455S), and glyceraldehyde 3-phosphate dehydrogenase (GAPDH, CST, 5174S) () were used for western blotting or immunohistochemistry (IHC). The secondary horseradish peroxidase (HRP)-conjugated antibody (8114S) was from CST. All other reagents and chemicals used in this study were of analytical grade.

### Preparing and phytochemically analysing WPL

WPL consists of nine herbs: Huang Qi (Guangdong Tiancheng Prepared Herbal Medicine Co., Ltd., China, 200501), Bai Zhu (Guangdong Zhixin traditional Chinese medicine Tabl Co., Ltd., China, 201101), Fu Ling (Guangdong Zhixin traditional Chinese medicine Tabl Co., Ltd., 201002), E Zhu (Guangdong medicinal materials co., China, E0520011), Tai Zi Shen (Guangdong medicinal materials co., T3520011), San Qi (KANGMEI, China, 201201201), Bai Hua She Cao (Guangdong medicinal materials co., B2020912), and Hou Gu Jun (Guangzhou Ziyunxuan Pharmaceutical Co., Ltd., China, 201001) Voucher specimens were deposited at the School of Pharmaceutical Sciences, Guangzhou University of Chinese Medicine (Guangzhou, China). All these herbs were extracted in water ten times the amount of the herbs and boiled twice for 2 h each. Next, San Qi and Hou Gu Jun powders were added and mixed. It was uniformly concentrated at 60 °C under reduced pressure, then vacuum-dried, and crushed to obtain 1 g of extract equivalent to 3.33 g of the crude drug. For in vivo management, WPL was dissolved in water (450 mg/mL).

WPL was dissolved in methanol (10 mg/mL), and a 5-μm filter or WPL solution was filtered by HPLC–MS/MS analysis to phytochemically examine the WPL components.

WPL was characterised using the negative and positive ionisation modes of TSQ Quantum Ultra-EMR HPLC–MS/MS (Thermo Scientific, USA). The operating parameters were as follows: an ion source applying ESI and an ion spray voltage of 3000 V in both positive and negative modes. For two modes, the low rate of sheath gas and the low rate of auxiliary gas were 30 mL/min and 10 mL/min, respectively, and the probe heater and capillary temperatures were set at 350 °C and 300 °C, respectively. The skimmer level was set to 1. Parallel reaction monitoring (PRM) scanning was performed to obtain MS data at a resolution of 70,000. Additionally, sample analyses were performed at 9, 17, and 18 normalised collision energies (NCE), and the MS detection resolution was 17,500. The TSQ Tune software Trace Finder (Thermo Scientific) was used to set up the instrument and process the quantitative data.

HPLC analysis was conducted on a Phenomenex C18 column (50 mm × 2.1 mm, 5 μm) with mobile phases A (acetonitrile) and B (0.1% formic acid) (v/v). The flow velocity was set to 1 mL/min, and the injection volume was 5 μL. A linear gradient (initial 0% A, 5% A 0–0.5 min; 5–95% A 0.5–1 min; 95% A 1.0–2.5 min; 95–5% A 2.5–3 min) was used for elution. The retention duration and HPLC–MS/MS data of WPL extracts and the reference compounds were compared to identify compounds, including notoginsenoside R1, calycosin-7-*O*-beta-d-glucoside, astragaloside A, and adenine-9-β-d-ribofuranoside.

Quantitative determination was performed by using six-point regression curves. Four reference compounds were used to prepare a stock solution diluted with methanol to a suitable concentration. HPLC–MS/MS was conducted to analyse the standard and WPL solutions. The peak area of each reference compound was calculated from the calibration curves. Compound contents in WPL were expressed as mg/g was and was calculated after the correlation between the peak area and the calibration curves of each analyte (Table 2).

**Table 2:** Quantitatively determined parameters of reference compounds in WPL

Analyte	Linearity range (μg/mL)	Regression equation Y = ax + b	r^2^
*Notoginsenoside R1*	0.000475–0.95	y = 11.58x − 1.269	0.9997
*Adenine-9-β-d-ribofuranoside*	0.422–13.5	y = 9.28E4x + 39.8	0.9994
*Astragaloside A*	22.82–456.3	y = 1.74E5x − 1.10E3	0.9999
*Calycosin-7-O-beta-d-glucoside*	4.00–120.0	y = 2.84E7x − 1.505E5	0.9995

### Animal experiments

Male BALB/c mice (2-month-old, 18–22 g) were purchased from the Laboratory Animal Centre of the Guangzhou University of Chinese Medicine. All mice were maintained at 23 ± 1 °C with sufficient water and food. The mice were randomly divided into five groups (n = 10 per group): vehicle control (H_2_O), MNNG (150 μg/mL), MNNG + WPL (3.75 g/kg/day and 15 g/kg/day), and MNNG + VitB12 (1 mg/kg). VitB12 was applied as the positive control for GPLs. The mice were administered an MNNG solution (150 μg/mL) with free drinking water for 6 weeks. After constructing the GPL mouse model by administering MNNG for 6 weeks, the respective groups orally received equal volumes of WPL, VitB12, or the vehicle for 8 weeks. The mice were sacrificed after the last administration, and stomach tissues were collected for further experiments. MNNG was dissolved in H_2_O to a concentration of 2 mg/mL and stored at 4 °C in the dark. This study was approved by the Institutional Animal Care and Use Committee of Guangzhou University of Traditional Chinese Medicine (NO.20201028003).

### Histological examination

The stomach tissues were fixed with 4% PFA in 0.1 mol/L phosphate buffer overnight, embedded in paraffin, and cut into 4-μm slices. Standard techniques were used to stain the sections with haematoxylin and eosin (H&E) to observe histological changes.

### Immunohistochemistry

Formalin-fixed, paraffin-embedded gastric mucosal tissues were selected to perform IHC. Sections were de-paraffinized and rehydrated with xylene and graded ethanol to water, followed by antigen retrieval and tissue blocking using 3% normal non-immune serum. Next, they were incubated with the primary antibodies IFN-γ (Abcam, ab9657, rabbit, polyclonal, 1/400) and CD4 (Abcam, ab203034, rabbit, polyclonal, 1/200) per the kit protocol (KGOS60, KeyGEN, Nanjing, China) at 4 °C overnight. After being washed with phosphate-buffered saline (PBS), the sections were incubated with HRP-conjugated secondary antibodies for 30 min at an ambient temperature. Tissue sections were stained with 3,3ʹdiaminobenzidine (DAB) and haematoxylin. The IHC results were observed under a microscope and analysed using Image-Pro Plus software (version 6.0; Media Cybernetics, Silver Spring, MD, USA) with the average combined optical density (IOD) to positive part (IOD/pixel) ratios.

### Western blotting

After preparation with radioimmunoprecipitation assay (RIPA) lysis buffer, protein samples were quantitated using a bicinchoninic acid (BCA) protein assay. Equal amounts of proteins were separated by sodium dodecyl-sulphate polyacrylamide gel electrophoresis (SDS-PAGE), transferred to polyvinylidene fluoride (PVDF) membranes which were blocked with 5% bovine serum albumin (BSA) solution, and incubated with primary antibodies at 4 °C overnight. The next day, the membrane was rinsed with Tris-buffered saline and Tween 20 (TBST), followed by incubation with HRP-conjugated secondary antibodies for 2 h at room temperature. Protein levels were detected using a chemiluminescence assay kit (Millipore, Massachusetts, USA). The density analysis of immunoblots was conducted using ImageJ software (NIH, USA).

### Quantitative reverse transcription PCR (RT-qPCR)

Total RNA was isolated from gastric mucosal tissues using a TRIzol reagent. Per the manufacturer’s protocol, we used the One-Step PrimeScript RT-PCR Kit to synthesise complementary DNA (cDNA) from total RNA. Next, qPCR was performed using ChamQTM Universal SYBR qPCR Master Mix (the reagents above were obtained from Vazyme Biotech Co., Ltd., Nanjing, China) and analysed on an ABI 7500 sequence detection system. The experiments were performed in triplicate, and the primer sequences were as follows: tumour necrosis factor alpha (TNF-α), forward 5′-AGGGTCTGGGC CATAGAACT-3′ and reverse 5′-CCACCACGCTCTTCTGTCTAC-3′; interleukin (IL)-6 (IL-6), forward 5′-ATGATGAGAAACGAGCCAATTG-3′ and reverse 5′-GCTTTGGCTTCTTTCTTACGAG-3′.

### Statistical analysis

All quantitative measures are described as the average ± standard deviation (SD). Two independent groups were compared using an unpaired *t*-test. Differences between more than two groups were compared using a one-way analysis of variance (ANOVA), followed by Dunnett’s post-hoc test. *P* < 0.05 was regarded as statistically significant. All data analyses were performed using GraphPad Prism 7.0 (GraphPad Software, San Diego, CA, USA). All experiments were conducted in triplicates.

## Results

### Screened WPL active compound and GPL targets

We obtained 282 active compounds and 6767 targets of WPL from the public databases. Based on a QED ≥ 0.3, 150 active compounds were identified. In addition, 127 active compounds and 758 targets were identified using a binomial statistical model. Additionally, 12 compounds and 43 targets of *Hericium erinaceus (Bull.) Pers* were retrieved from the HIT database. Furthermore, 78 and 73 targets of calycosin 7-*O*-glucoside and notoginsenoside R1 were collected from the PharmMapper platform, respectively. After merging all the results and removing duplicate values, 141 active compounds and 854 targets of WPL were identified.

A total of 1645 GPL targets were screened from the GeneCards database, and 632 targets were further screened with relevance scores ≥ 5.

### PPI network analysis

**Fig. 1:**
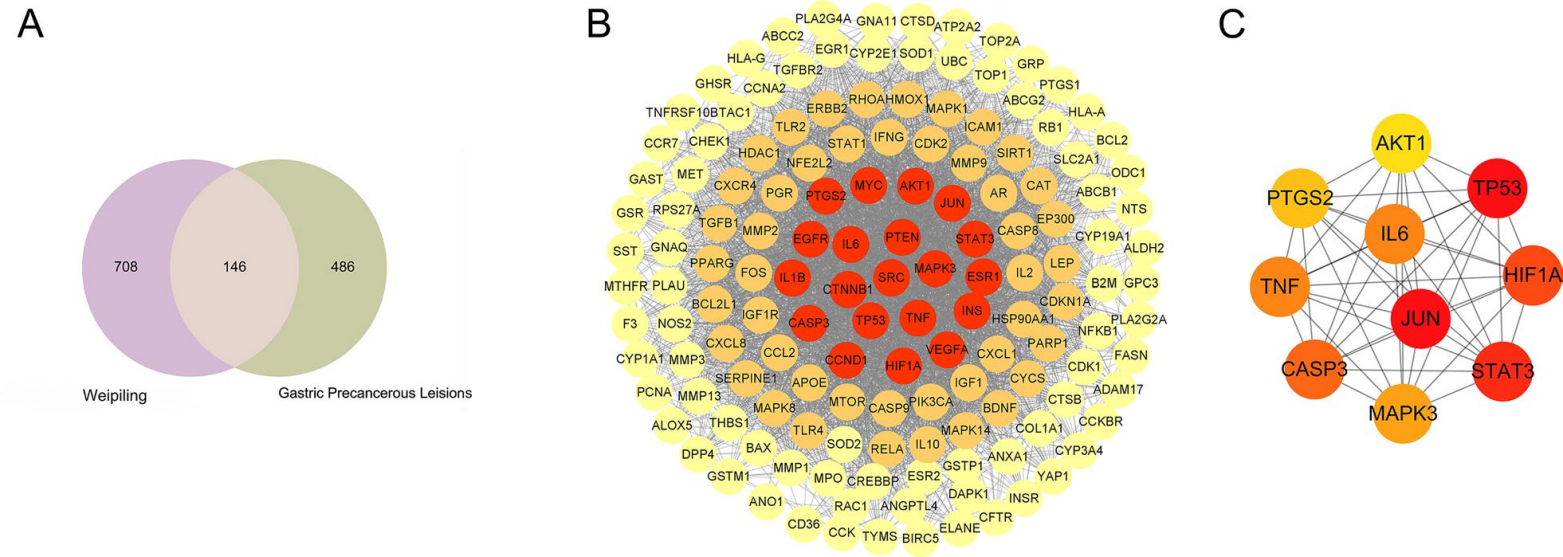
Anti-gastric precancerous lesions (GPLs) and protein–protein interaction (PPI) network of potential weipiling (WPL) targets. **A** Venn diagram of 146 common targets. **B** The PPI network of 146 targets was constructed using the STRING database and Cytoscape software 3.8.0. **C** The top ten hub targets were selected from the PPI network (**B**)

The Venny 2.1 tool was used to identify the common WPL and GPL targets, and 146 were retrieved (Fig. 1A). Then, 110 active compounds of WPL were identified using the 146 common targets. PPI network analysis was performed using the STRING database, with 146 common targets (Fig. 1B). The network consisted of 146 nodes and 3713 edges, and the average degree value was 50.9. CytoHubba, a Cytoscape plug-in, was used to select hub targets. The hub target network with ten nodes and 45 edges was visualised using Cytoscape software (Fig. 1C). The top ten hub targets were AKT1, PTGS2, TNF, CASP3, MAPK3, STAT3, HIF1A, TP53, IL6, and JUN.

### GO and KEGG enrichment analyses

**Fig. 2:**
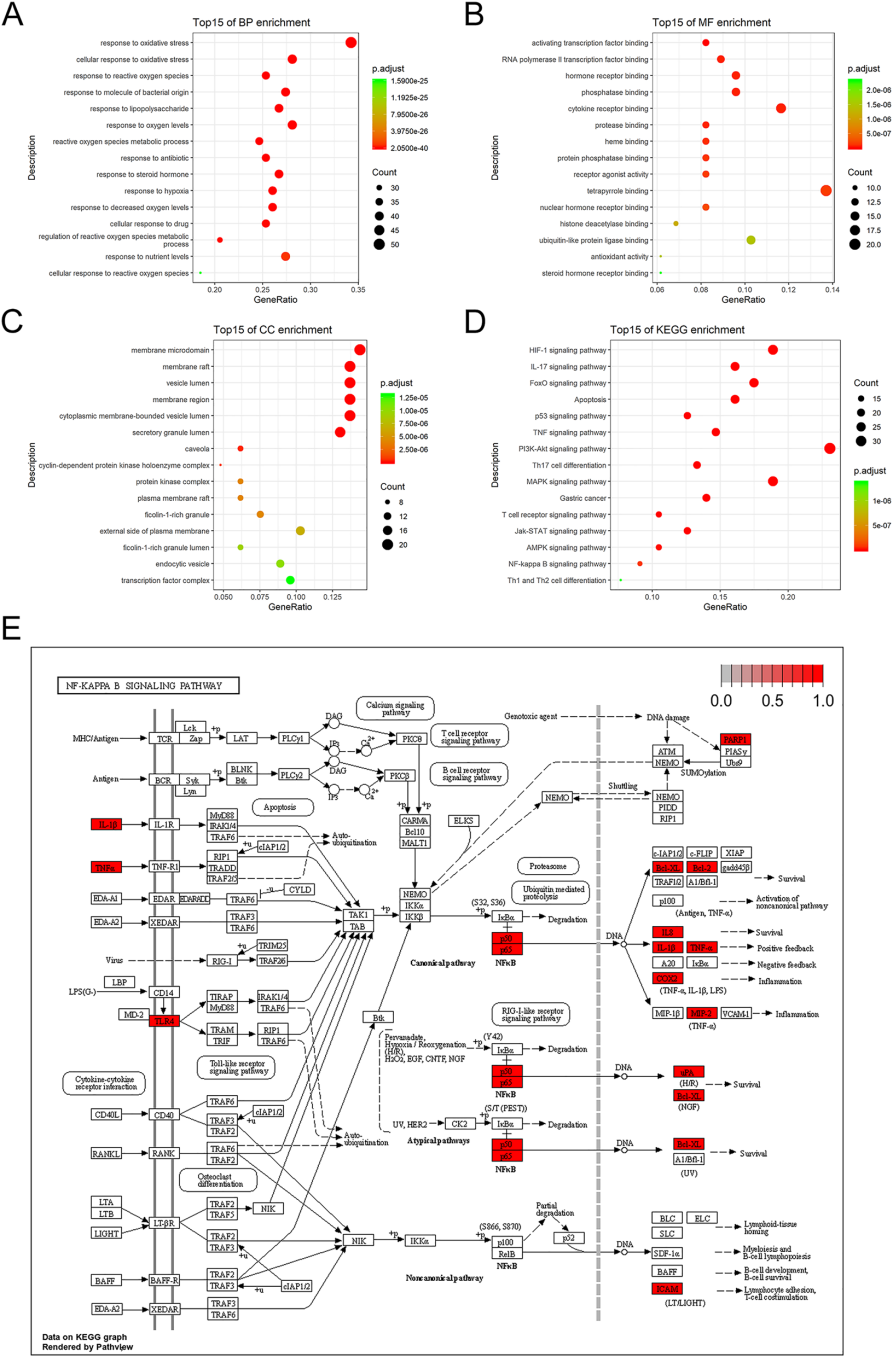
Gene Ontology (GO) and Kyoto Encyclopaedia of Genes and Genomes (KEGG) enrichment analyses. **A** Top 15 biological process (BP) enrichment items. **B** Top 15 molecular function (MF) enrichment items. **C** Top 15 cellular components (CC) enrichment items. **D** Top 15 KEGG enrichment items. **E** Map of the NF-κB signalling pathway. Red boxes represent the key targets

We obtained 1759 GO items, including 72 molecular function (MF) items, 1627 biological process (BP) items, and 60 cellular components (CC) items, with screening criteria of *P* < 0.01. Figure 2A–C show the top 15 MF, BP, and CC items. The MF results suggested that WPL mainly focused on activating transcription factor binding, RNA polymerase II transcription factor binding, and hormone receptor binding. The BP items mainly included responses to oxidative stress, cellular responses to oxidative stress, and responses to reactive oxygen species (ROS). CC items mainly included membrane microdomains, membrane rafts, and vesicle lumens. KEGG enrichment analysis showed that anti-GPL WPL was mainly involved in 165 signalling pathways (*P* < 0.01). The top 15 enriched pathways were the hypoxia-inducible factor (HIF)-1, IL-17, and NF-κB signalling pathways (Fig. 2D–E).

### Herb-compound-target (H-C-T) and target-pathway (T-P) network analysis

**Fig. 3:**
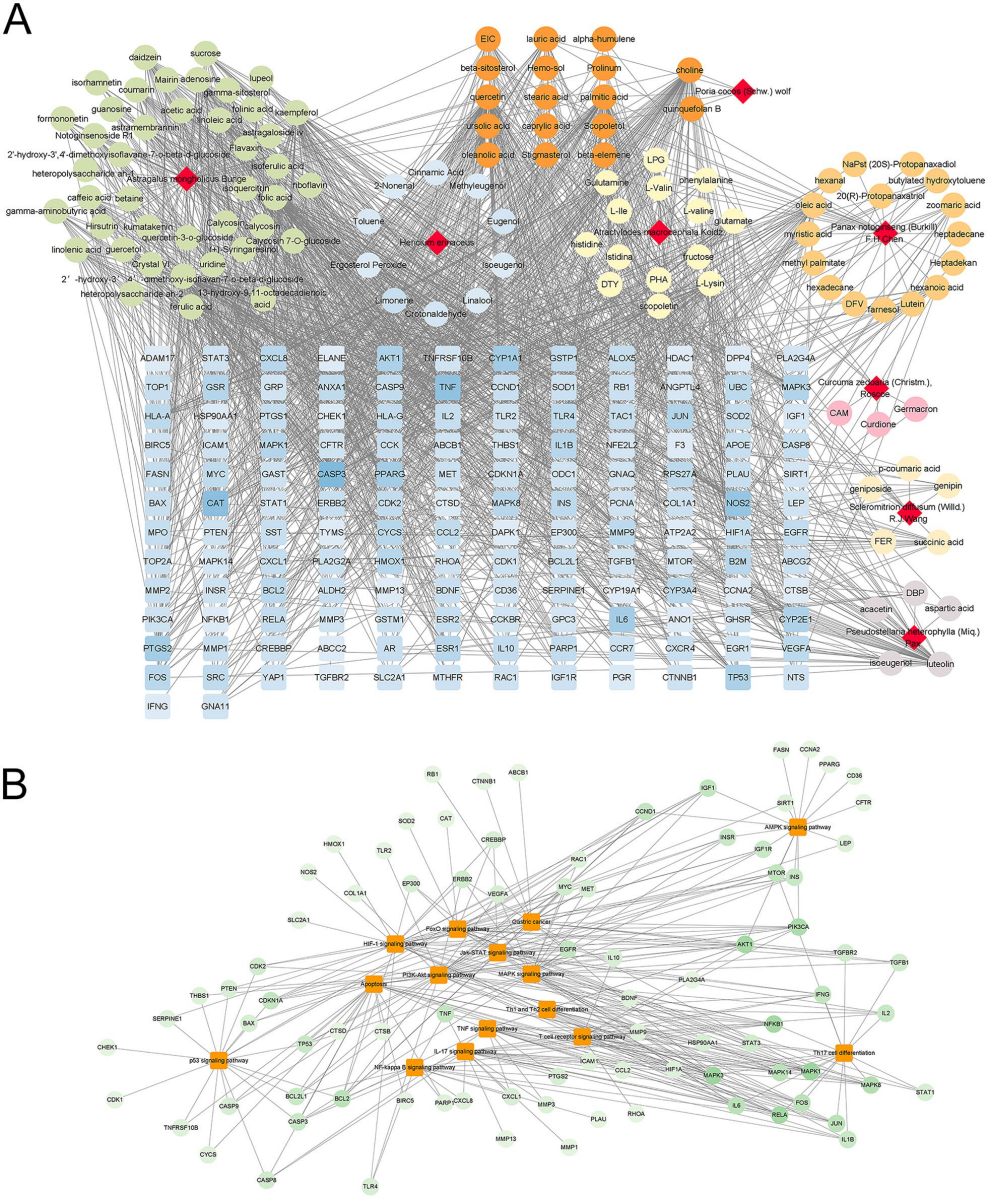
Herb-compound-target (H-C-T) and target-pathway (T-P) network analysis. **A** The H-C-T and **B** T-P networks were built using Cytoscape software

The H-C-T and T-P networks of WPL against GPLs were constructed using Cytoscape software (Fig. 3). The H-C-T network is composed of 266 nodes and 1469 edges. Herbs, compounds, and targets are represented by prisms, circles, and squares, respectively. In addition, the TP network includes 102 nodes and 308 edges. The targets and the top 15 KEGG pathways are represented by circles and squares, respectively. These networks suggest that WPL-treated GPLs involve multiple components, targets, and pathways.

### Molecular docking analysis

**Fig. 4:**
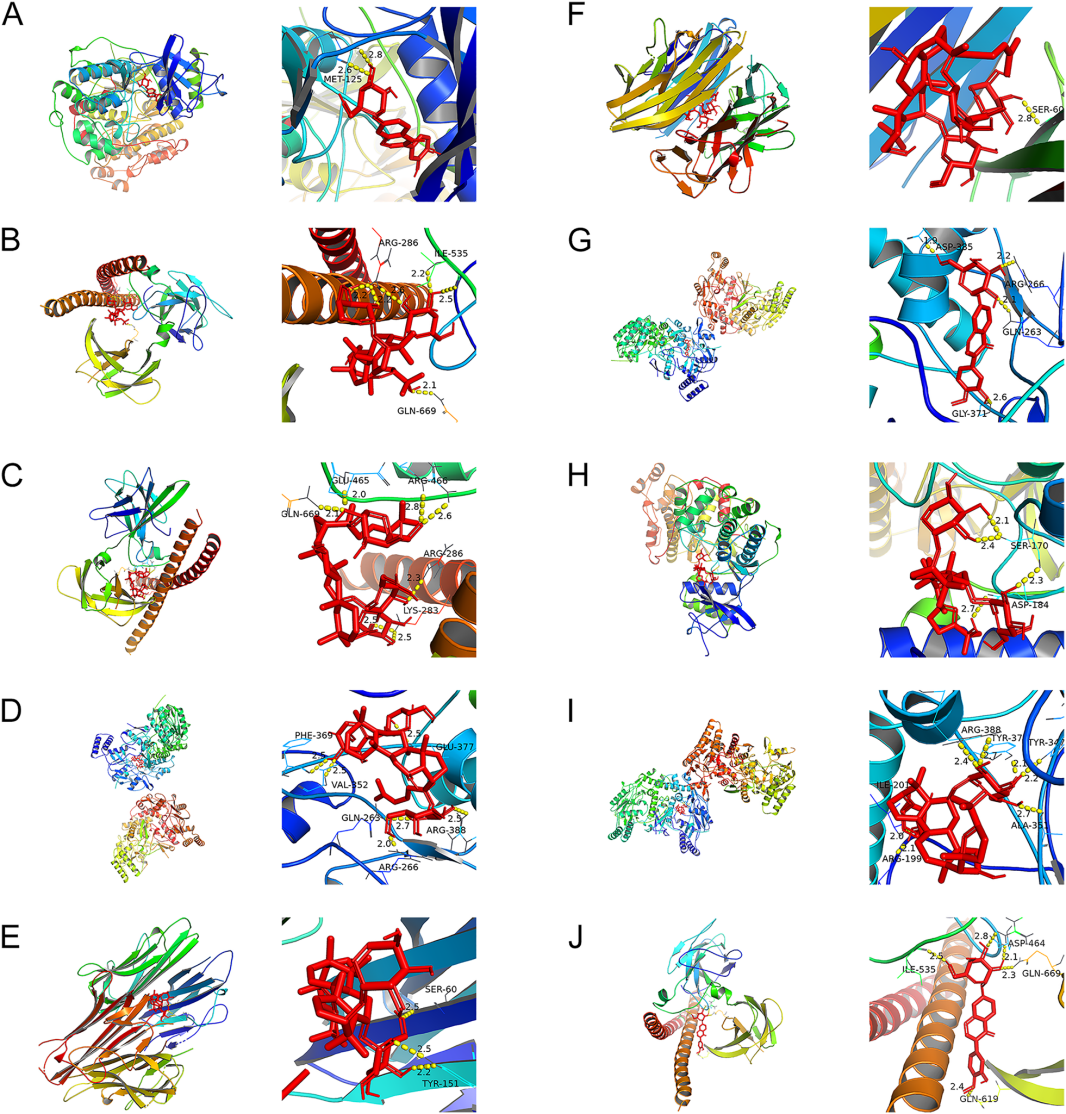
Docking of WPL-related active compounds and top hub genes. **A** Molecular docking of calycosin-7-*O*-β-d-glucoside and MAPK3, **B** astragaloside IV and JUN, **C** notoginsenoside R1 and JUN, **D** notoginsenoside R1 and NOS2, **E** astragaloside IV and TNF, **F** notoginsenoside R1 and TNF, **G** calycosin-7-*O*-β-d-glucoside and NOS2, **H** astragaloside IV and MAPK3, **I** astragaloside IV and NOS2, and **J** calycosin-7-*O*-β-d-glucoside and JUN

In our study, we docked seven hub targets (JUN, TNF, TP53, CASP3, MAPK3, NOS2, and BCL2) with four active compounds (calycosin-7-*O*-β-d-glucoside, astragaloside IV, notoginsenoside R1, and quinquefolan B) (Table 3) of WPL. Based on affinity < − 5 kcal/mol, we obtained 27 pairs of docking results (Table 4). Moreover, calycosin-7-*O*-β-d-glucoside had a good binding ability with MAPK3 (− 9.1 kcal/mol), NOS2 (− 7.9 kcal/mol), and JUN (− 7.5 kcal/mol). Astragaloside IV had a good binding ability with JUN (− 8.7 kcal/mol), TNF (− 8.2 kcal/mol), MAPK3 (− 7.7 kcal/mol), and NOS2 (− 7.7 kcal/mol). Notoginsenoside R1 had a good binding ability with JUN (− 8.2 kcal/mol), NOS2 (− 8.2 kcal/mol), and TNF (− 8 kcal/mol) (Fig. 4).

**Table 3:** Chemical structures of WPL active compounds

Synonyms	Cas	Molecular Formula	2D Structure
Notoginsenoside R1	80418-24-2	C47H80O18	
Calycosin-7-*O*-β-d-glucoside	20633-67-4	C22H22O10	
Astragaloside IV	84687-43-4	C41H68O14	
Quinquefolan B	109767-06-8	C10H13N5O4

**Table 4:** Molecular docking results

Chem	GENE	PDB	Best affinity
Calycosin-7-*O*-β-d-glucoside	MAPK3	4qtb	− 9.1
Astragaloside IV	JUN	1s9k	− 8.7
Notoginsenoside R1	JUN	1s9k	− 8.2
Notoginsenoside R1	NOS2	3e7g	− 8.2
Astragaloside IV	TNF	2az5	− 8.2
Notoginsenoside R1	TNF	2az5	− 8
Calycosin-7-*O*-β-d-glucoside	NOS2	3e7g	− 7.9
Astragaloside IV	MAPK3	4qtb	− 7.7
Astragaloside IV	NOS2	3e7g	− 7.7
Calycosin-7-*O*-β-d-glucoside	JUN	1s9k	− 7.5
Notoginsenoside R1	BCL2	2w3l	− 7.2
Notoginsenoside R1	TP53	3q05	− 7.1
Quinquefolan B	MAPK3	4qtb	− 7.1
Calycosin-7-*O*-β-d-glucoside	CASP3	1gfw	− 7.1
Astragaloside IV	CASP3	1gfw	− 7.1
Quinquefolan B	NOS2	3e7g	− 6.9
Calycosin-7-*O*-β-d-glucoside	TP53	3q05	− 6.9
Quinquefolan B	TNF	2az5	− 6.8
Astragaloside IV	TP53	3q05	− 6.7
Astragaloside IV	BCL2	2w3l	− 6.7
Notoginsenoside R1	CASP3	1gfw	− 6.6
Calycosin-7-*O*-β-d-glucoside	TNF	2az5	− 6.6
Quinquefolan B	JUN	1s9k	− 6.4
Calycosin-7-*O*-β-d-glucoside	BCL2	2w3l	− 6.3
Quinquefolan B	CASP3	1gfw	− 6.1
Notoginsenoside R1	MAPK3	4qtb	− 6
Quinquefolan B	TP53	3q05	− 5.4

### Quantitative analysis of the main components in WPL

**Fig. 5:**
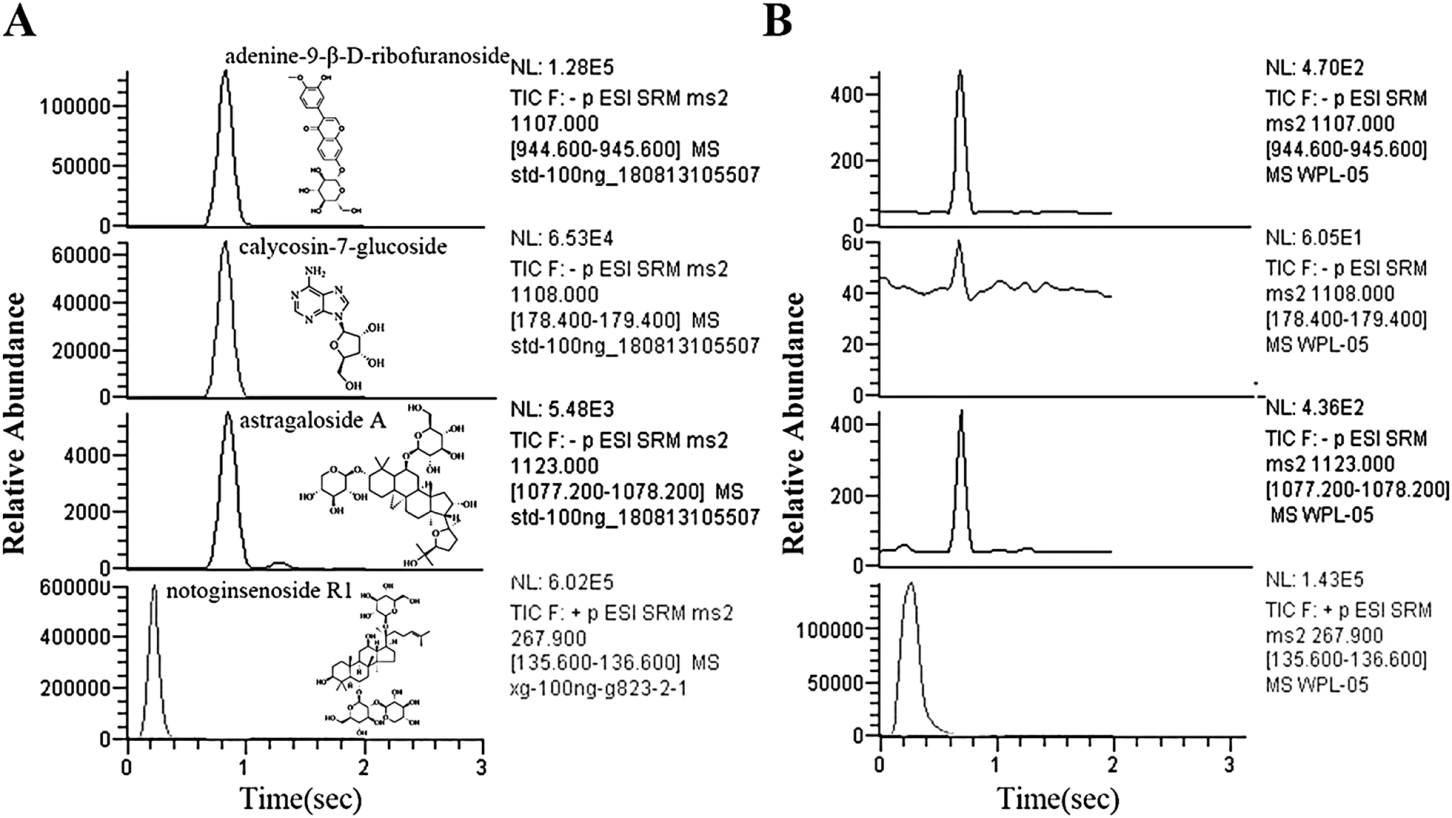
Extracted ion chromatograms to quantify the analytes in WPL. **A**, **B** Ion chromatograms realizing the highest intensity in the full MS/MS spectrum of precursor ions were extracted from the normative (**A**) and WPL solutions, (**B**) respectively

The main components of WPL were determined by HPLC–MS analysis by matching the retention duration and mass spectra of the normative compounds with those of WPL. The retention duration and ion chromatograms were compared with those of the reference compounds to identify the main components of WPL, including notoginsenoside R1, calycosin-7-*O*-beta-d-glucoside, astragaloside A, and adenine-9-β-d-ribofuranoside (Fig. 5, Table 5). Additionally, notoginsenoside R1, calycosin-7-*O*-beta-d-glucoside, astragaloside A, and adenine-9-β-d-ribofuranoside were detected at m/z 929.0, m/z 447.0, m/z 785.0, and m/z 267.9 at the [M^+^H]^+^ mode and at m/z 324.8, m/z 284.9, m/z 324.9, m/z 136.1 at the [M^−^H]^–^ mode, respectively, in high-resolution MS/MS configured with ESI (Table 5). Quantification was then performed by extracting the fragment ions with the highest intensity in the full MS spectra of the precursor ion (Fig. 5, Table 5). A standard calibration curve was used to determine the peak areas of the analytes. The calibration curves of all reference compounds displayed good linearity, which could be used to calculate the amount of each compound in WPL (*r*^2^ ≥ 0.9994, Table 2). Notoginsenoside R1 and calycosin-7-glucoside were the most abundant compounds (Table 2), indicating that these compounds should be regarded as chemical markers of WPL.

**Table 5:** Retention time, precursor and quantitative ion m/z, normalized collision energy, and analyte content in WPL

Analyte	Retention time (s)	Precursor ion (m/z)	Quantitative fragment (m/z)	Normalized collision energy (NCE, %)	Content (mg/g)
*Notoginsenoside R1*	53	929.0	324.8	17	1.524
*Adenine-9-β-D-ribofuranoside*	14	267,9	136.1	17	0.448
*Astragaloside A*	51	785.0	324.9	9	0.139
*Calycosin-7-O-beta-d-glucoside*	50	447.0	284.9	18	1.474

### WPL inhibits MNNG-induced chronic gastric inflammatory injury and attenuates inflammation-mediated gastric mucosa tissue damage

**Fig. 6:**
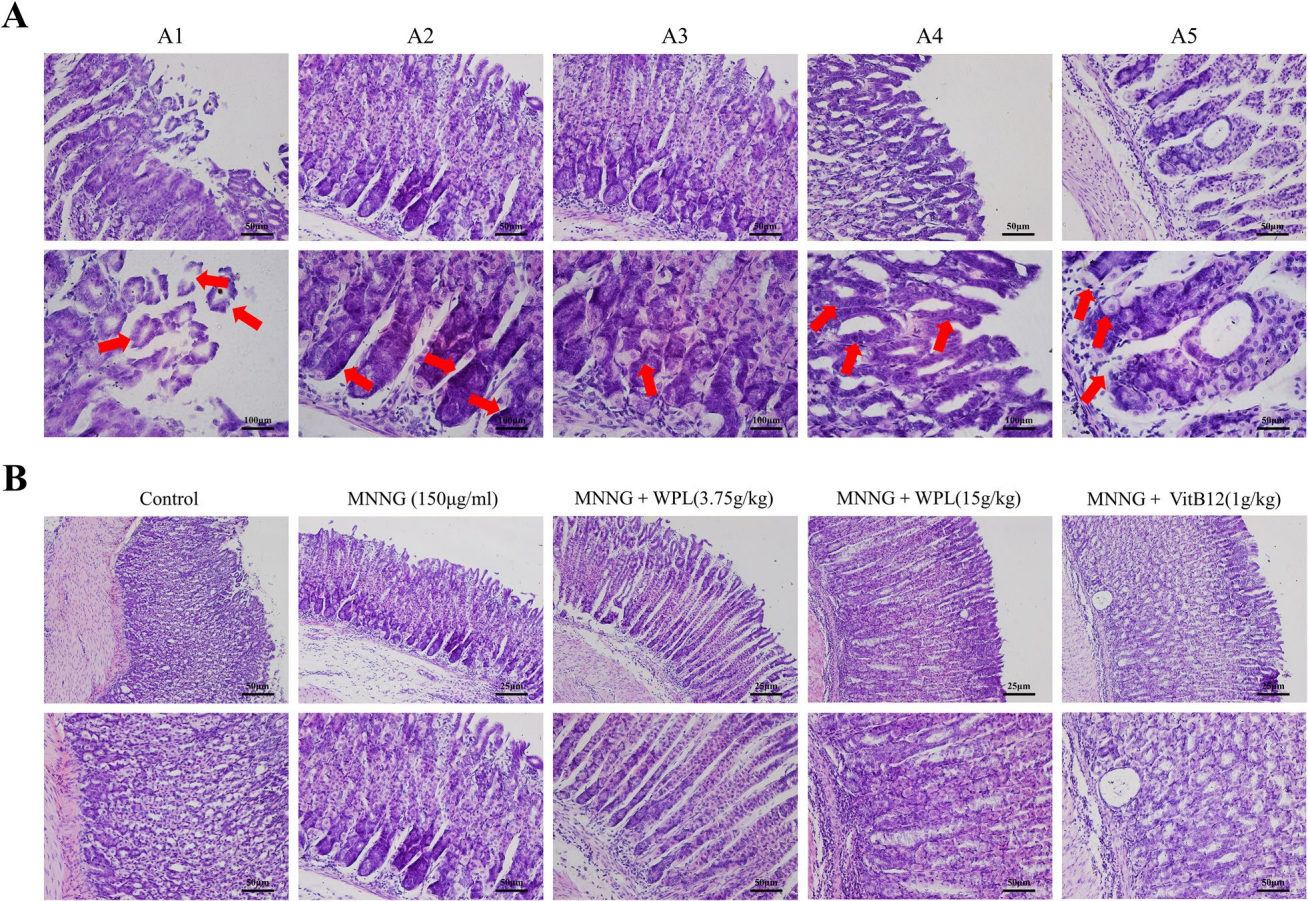
WPL suppresses *N*-methyl-*N*-nitro-*N*-nitrosoguanidine (MNNG)-induced chronic gastric inflammatory injury and attenuates inflammation-mediated gastric mucosa tissue damage. **A1**–**A5** show the significant structure lesion of the model group by H&E staining: **A1** shedding of epithelium, **A2** inflammatory infiltration, **A3** cavity fusion of gastric mucosa, **A4** intestinal metaplasia (IM), **A5** and dysplasia cells. **B** Representative images provide details about the effect of WPL using H&E staining

GPLs were induced using MNNG [43] to determine the anti-inflammatory role of WPL. Details regarding the effects of WPL were provided by H&E staining. According to Fig. 6A, the model group showed significant structural lesions of the gastric mucosa, such as epithelial shedding, and inflammatory infiltration. Likewise, epithelial cells exhibited vacuolation and cavity fusion in the gastric mucosa. Physaliphorous cells with a symmetrical thickness of the basement membrane were also visible. Dysplastic gastric epithelial cells were observed with enlarged, hyperchromatic, and crowded nuclei in the basement of the membrane (dysplasia). Furthermore, the irregular glands partially bifurcated or branched, indicating IM and dysplasia development. All these morphological changes suggested that apparent GPLs were present in the gastric mucosa (Fig. 6B). In contrast, the gastric mucosa in the WPL and VitB12 groups exhibited less epithelial shedding, ulceration, and leukocyte infiltration. In addition, the gastric mucosa from the WPL group showed a more regular glandular cavity morphology and less or fewer enlarged, hyperchromatic, and crowded nuclei, indicating that WPL ameliorated the MNNG-induced gastric mucosal lesions.

**Fig. 7:**
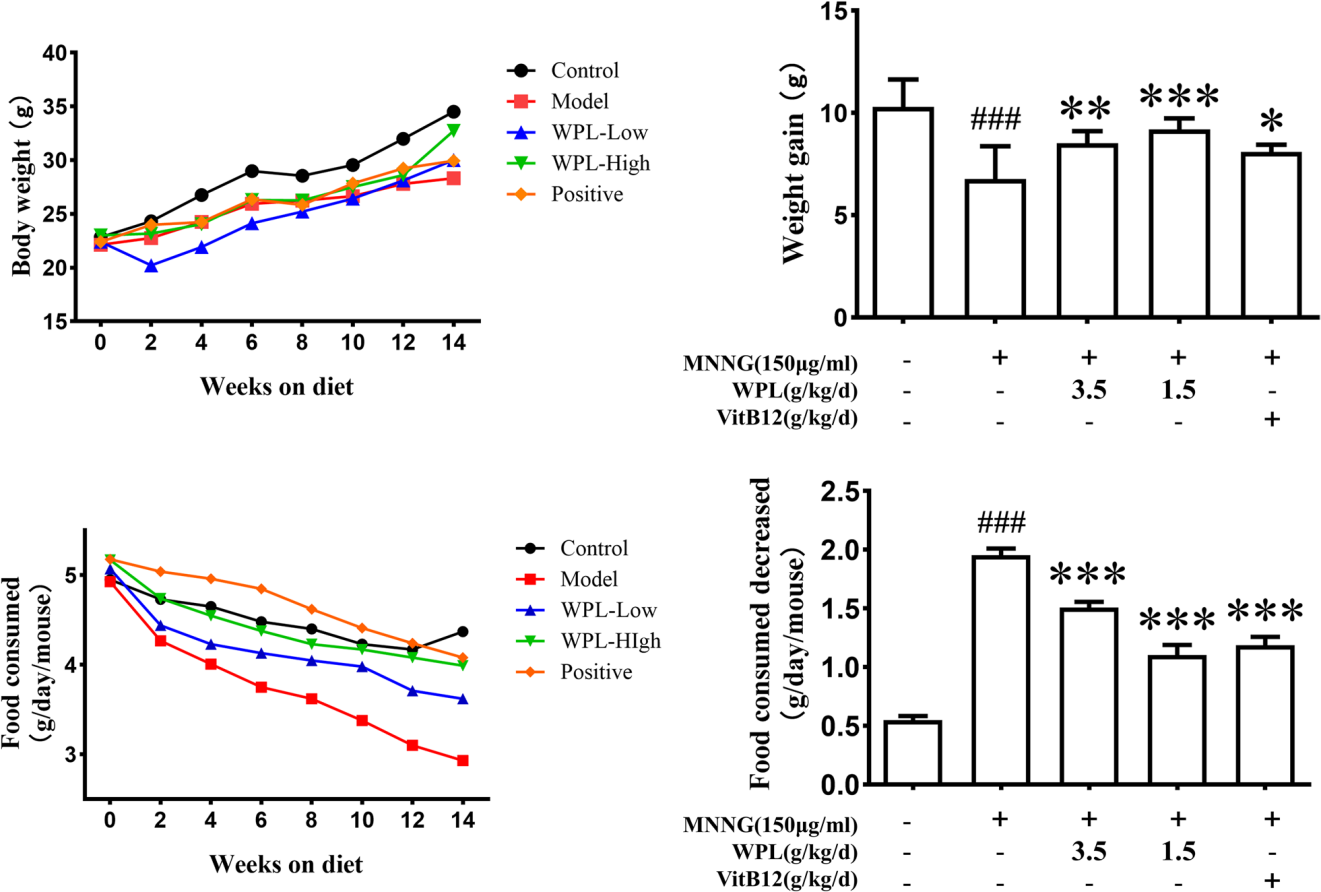
WPL moderately attenuates the weight loss and food intake (FI) reduction caused by MNNG. **A** and **C** show the body weight (BW) gain and food consumption tendencies. **B** and **D** show the decrease in BW and food consumed. Data are described as the means ± standard deviation (SD) from three separate experiments. ^###^P < 0.001 versus the control group. *P < 0.05, **P < 0.01, ***P < 0.01 versus the MNNG-treated group

Body weight (BW) and food intake (FI) were measured every 2 weeks to determine the gastric protective effects of WPL (Fig. 7). The BW gain rate and FI of the MNNG group mice significantly declined. However, administering WPL or VitB12 increased the BW and FI of mice, indicating that WPL and VitB12 moderately attenuated the weight loss and FI reduction caused by MNNG.

### WPL inhibits pro-inflammatory T helper 1 (Th1) cell infiltration to ameliorate gastric damage

**Fig. 8:**
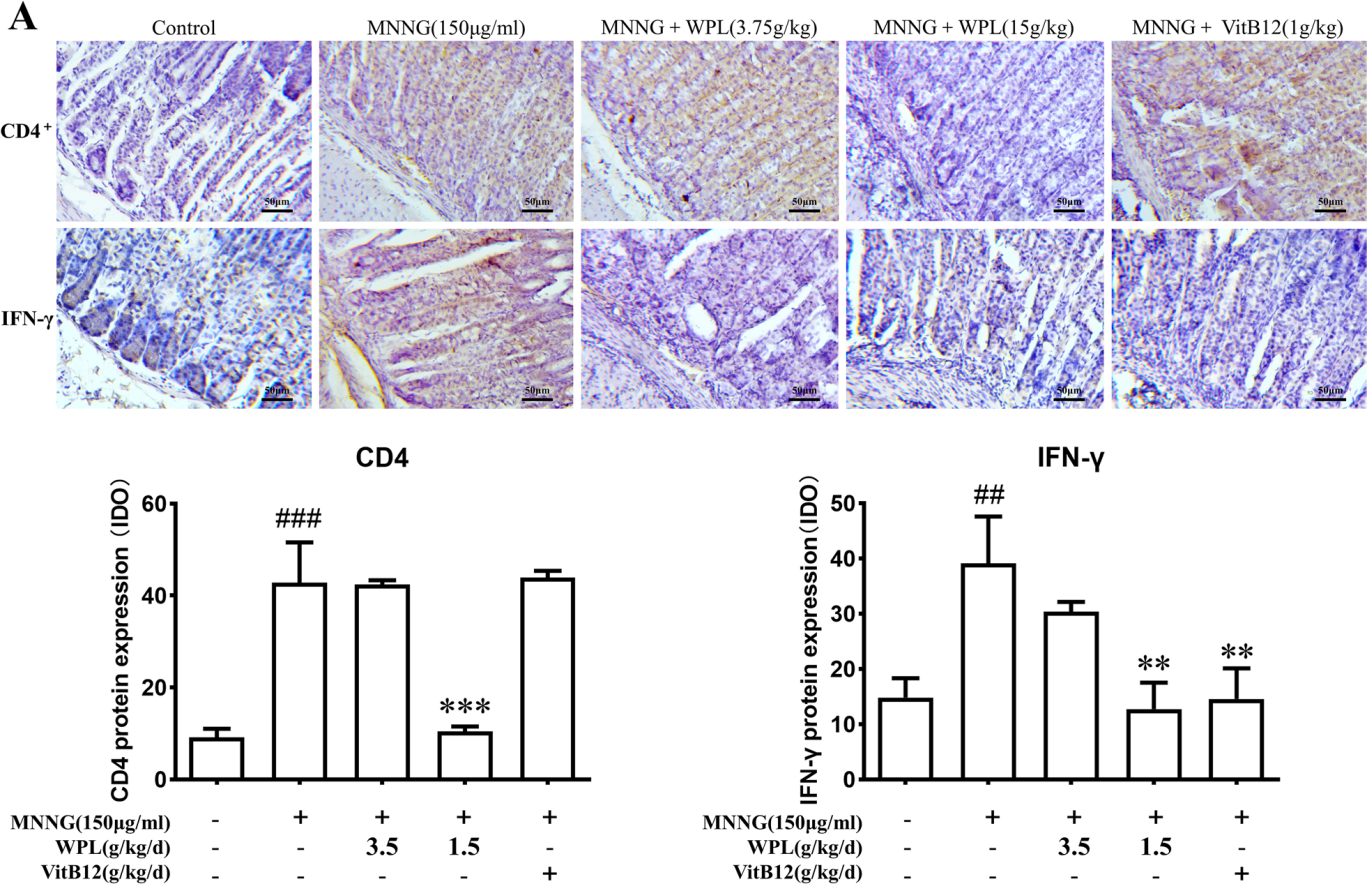
WPL inhibits pro-inflammatory T helper 1 (Th1) cell infiltration to ameliorate gastric damage. **A** shows the Th cells (the first row), including Th1 cell (the second row) infiltration in the inflammatory gastric mucosa tissue by immunohistochemistry (IHC) staining. **B** Optical density analysis was used to quantify relative protein levels by applying ImageJ software. Data are described as the means ± SD in three separate experiments. ^##^P < 0.01, ^###^P < 0.001 versus the control group. **P < 0.01, ***P < 0.01 versus the MNNG-treated group. Bar = 100 μm

IHC was performed to examine Th cell infiltration in inflammatory gastric mucosa tissues to uncover the pro-inflammatory effects of Th cells on GPL development. Abundant Th cell (CD4^+^) infiltration was observed after administering MNNG (Fig. 8A, first row). WPL and VitB12 inhibited Th cell infiltration, and a remarkable reduction was observed in the WPL group (*P* < 0.01). Among the Th cell subsets, Th1 (IFN-γ^+^) cells are pro-inflammatory factors that drive the growth of GPLs [9, 44, 45]. Based on the infiltration of total Th cells in the gastric mucosal tissues of the MNNG group (Fig. 8A, first row), the number of Th1 (IFN-γ^+^) cells significantly increased in the model group (Fig. 8A, second row, *P* < 0.01). Interestingly, both WPL and VitB12 suppressed excess pro-inflammatory Th1 cell infiltration (Fig. 8A, B) (*P* < 0.01).

### WPL decoction showed inhibitory effects on NF-κB signalling pathway and decreased pro-inflammatory enzymes and cytokine production in the gastric mucosa

**Fig. 9:**
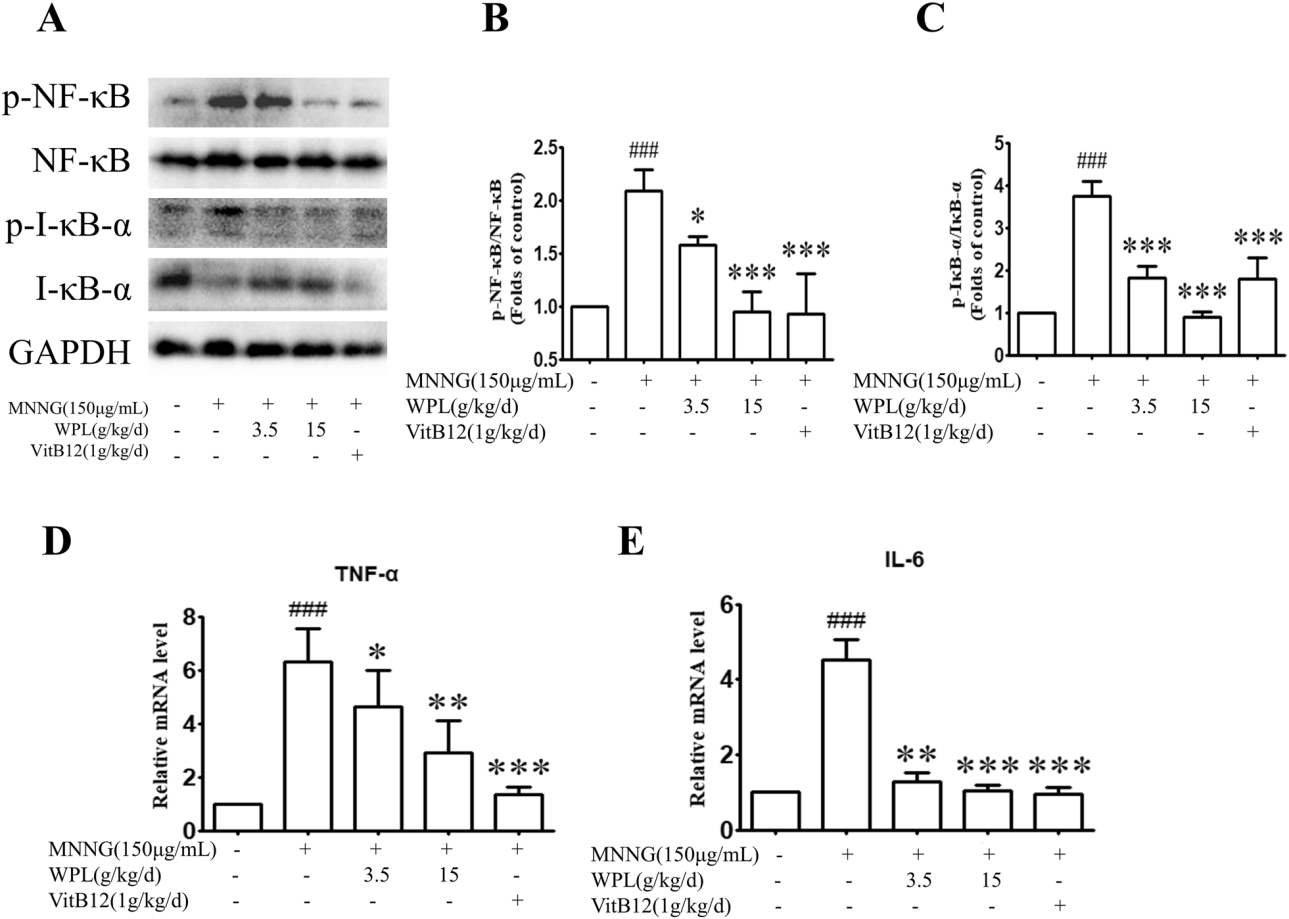
WPL has inhibitory roles in the nuclear factor kappa B (NF-κB) signalling path and decreases pro-inflammatory mediator generation in the gastric mucosa. **A** Western blotting was used to analyse NF-κB p65, phospho-NF-κB p65, IκB-α, and phospho-IκB-α protein levels, and glyceraldehyde 3-phosphate dehydrogenase (GAPDH) was applied as a loading control. **B**, **C** Densitometry analysis was adopted to quantify relative protein levels using ImageJ software. **D**, **E** Real-time PCR analysis was adopted to detect the relative mRNA levels of tumour necrosis factor alpha (TNF-α) and IL-1β in the gastric mucosa. ^###^P < 0.001 versus the control group. *P < 0.05, **P < 0.01, ***P < 0.01 versus the MNNG-treated group

**Fig. 10:**
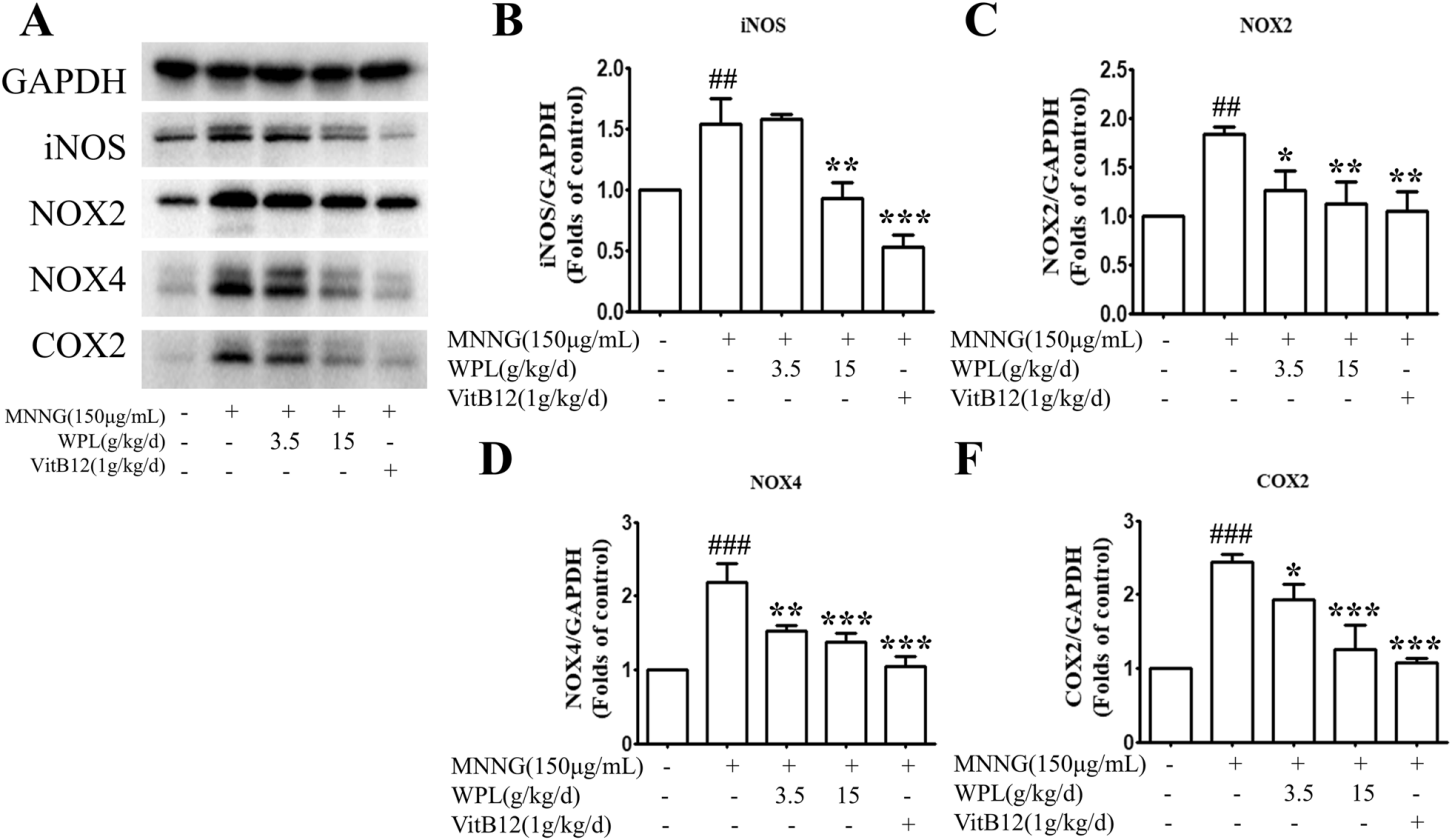
WPL attenuates MNNG-induced generation of inflammatory mediators in the gastric mucosa. **A** Inducible nitric oxide synthase (iNOS), cyclooxygenase-2 (COX-2), nicotinamide adenine dinucleotide phosphate oxidase (NADPH oxidase (NOX))2, and NOX-4 protein levels in the gastric mucosa were analysed by western blotting and GAPDH was applied as an internal control. **B**–**E** Densitometry analysis was adopted to quantify relative protein levels using ImageJ software. ^##^P < 0.01, ^###^P < 0.001 versus the control group. **P < 0.01, ***P < 0.01 versus the MNNG-treated group

As the results of KEGG enrichment analysis, NF-κB signalling is critical for gastric inflammation and GPL development. Exposure to MNNG for 14 weeks significantly enhanced NF-κB and IκBα phosphorylation. Nevertheless, MNNG-induced NF-κB and IκB-α phosphorylation was markedly reversed by the WPL pre-treatment (Fig. 9A–C). Compared with VitB12, WPL showed a comparable inhibitory effect on the NF-κB signalling pathway. Additionally, MNNG markedly induced transcriptional TNF-α and IL-6 upregulation, whereas WPL inhibited these effects (Fig. 9D, E), indicating that WPL can inhibit MNNG-induced inflammation in the gastric mucosa. Inflammatory stimuli can activate various enzymes, such as iNOS and COX-2, to promote inflammation. NF-κB signalling frequently induces NOX protein family members, such as NOX-2 and NOX-4, to complicate the inflammatory response by generating ROS. In our study, MNNG increased iNOS and COX-2 protein levels, whereas the WPL pre-treatment markedly decreased these protein levels (Fig. 10A–C). Furthermore, WPL attenuated MNNG-induced NOX2 and NOX4 protein upregulation (Fig. 10A, D, E). Therefore, WPL exerts anti-inflammatory and gastric mucosa-protective effects by inhibiting NF-κB signalling and pro-inflammatory enzyme expression.

## Discussion

The poor outcome and mortality of GC can be prevented by early diagnosis and intervention at the GPL stage [46]. As a compound Chinese medicine and based on the theories of traditional Chinese medicine, WPL can ameliorate GPLs by enhancing the spleen, dispersing blood stasis, and detoxification. According to previous clinical trials, WPL has a great capacity to attenuate the clinical symptoms of GPLs [47]. In this study, network pharmacological analysis suggested that WPL relieves GPLs and may be associated with antioxidative stress and anti-inflammatory effects. An in vivo assay revealed that WPL attenuated MNNG-induced GPLs by inhibiting NF-κB pathway activation and pro-inflammatory Th1 cell infiltration in the gastric mucosa.

Here, we retrieved 110 active compounds and 146 potential targets of anti-GPL WPL from public databases and references. Notoginsenoside R1, calycosin-7-*O*-beta-d-glucoside, astragaloside IV, and adenine-9-β-d-ribofuranoside were selected as chemical markers owing to their commercial value, high content, and pharmacological activity. HPLC–MS/MS results showed that notoginsenoside R1, with a content of 1.524 mg/g, was one of the most abundant compounds in WPL through quantitative analysis of the chemical markers. Notoginsenoside R1 exhibits pharmacological activities in alleviating oxidative stress, eliminating inflammation, and protecting damaged cells [7], suggesting that WPL extracts can be used to treat inflammatory diseases.

KEGG enrichment analysis showed that inflammation-related signalling pathways, including the HIF-1, IL-17, and NF-κB signalling pathways, were involved in anti-GPL WPL. GPL and GC development are related to continuous inflammation of the gastric mucosa, including NF-κB-mediated inflammation, the production of pro-inflammatory cytokines, and the infiltration of pro-inflammatory cells [48, 49]. Study suggests that NF-κB may promote tumorigenesis by regulating inflammation, cell proliferation, and apoptosis. And the activation of NF-κB contributes to GPL [50]. In our study, we found that phosphorylation expression of NF-κB and IκB-α was upregulated in MNNG-induced GPL mice, which was weakened by WPL addition. Therefore, the traditional Chinese medicine, WPL, may serve as an inhibitor of the NF-κB pathway. For example, extracts from *Astragalus mongholicus Bunge* decreased angiogenesis-related molecules, such as vascular endothelial growth factor and COX-2, in ovarian tumor-bearing mice. Compounds extracted from *Atractylodes lancea (Thunb.) DC*, such as beta-eucalyptol and atractylone*,* can inhibit inflammation by blocking the NF-κB signalling pathway [20–22]. A previous study also demonstrated that the most effective ingredients of *Panax notoginseng (Burkill) F. H. Chen* had various pharmacological functions, such as anti-oxidative stress, anti-inflammatory, and anti-apoptotic effects [51, 52]. *Scleromitrion diffusum (Willd) R. J. Wan*g. and *Hericium erinaceus (Bull.) Pers.* are commonly used to treat gastrointestinal diseases, such as GC and CAG, by inhibiting tumour angiogenesis, proliferation, and apoptosis [24, 28–31]. Our previous study showed precancerous changes in the gastric mucosa in MNNG-induced GPL rats [7]. The most significant finding of this study was that WPL significantly attenuated MNNG-induced GPLs by inhibiting IκB/NF-κB pathway activation and pro-inflammatory Th1 cell infiltration in the gastric mucosa.

The NF-κB signalling pathway is upstream of NOX2 and NOX4. NOX2 and NOX4 upregulation indicated ROS generation in MNNG-treated mice, and WPL reduced NOX2 and NOX4 levels. COX-2 and iNOS are downstream enzymes of the NF-κB pathway, and the products catalysed by these two enzymes are PEG2 and NO, which are further metabolised into ROS and RNS. An inhibitory effect of WPL was observed in this pathway. Moreover, cytokines and their function in recruiting pro-inflammatory Th1 cells potentiate WPL’s efficacy. WPL reversed the pro-inflammatory effects of Th1 cell infiltration and INF-γ secretion. MNNG promoted TNF-α and IL-6 transcriptional levels, implying that the nuclear translocation of NF-κB is induced in the gastric mucosa. However, WPL exerted inhibitory effects on TNF-α and IL-6 transcription, suggesting that it may inhibit the nuclear translocation of NF-κB.

## Conclusion

In summary, the present study demonstrated that WPL inhibits MNNG-induced gastric inflammation and ameliorates inflammatory injury by suppressing the excess pro-inflammatory Th1 cell infiltration, inhibiting NF-κB signalling activation, and attenuating the expression of inflammatory enzymes in vivo. Our study provides evidence that WPL has a beneficial role in the intervention of GC formation and development. Unfortunately, our study also has shortcomings, such as WPL treatment of GPLs involves multiple pathways, we only validated NF-κB signalling pathway in animal study. Other mechanisms will be the focus of our follow-up study.

## Data Availability

The datasets used and/or analysed during the current study are available from the corresponding author on reasonable request.

## Abbreviations

WPL: Weipiling
GPLs: Gastric precancerous lesions
HPLC–MS/MS: High-performance liquid chromatography with tandem mass spectrometry
MNNG: *N*-Methyl-*N*-nitro-*N*-nitrosoguanidine
H&E: Haematoxylin and eosin
Th1: T helper 1
INF-γ: Interferon-gamma
IHC: Immunohistochemistry
NF-κB: Nuclear factor kappa B
RT-qPCR: Reverse transcription polymerase chain reaction
iNOS: Inducible nitric oxide synthase
COX-2: Cyclooxygenase-2
NADPH oxidase (NOX): Nicotinamide adenine dinucleotide phosphate oxidase
GC: Gastric cancer
CAG: Chronic atrophic gastritis
IM: Intestinal metaplasia
NOCs: *N*-Nitroso compounds
TCMID: Traditional Chinese Medicine Integrated Database
TCMSP: Traditional Chinese Medicine Systems Pharmacology Database and Analysis Platform
STITCH: Search Tool for Interacting Chemicals
QED: Quantitative estimate of drug-likeness
HIT: Herb Ingredients’ Targets
PPI: Protein–protein interaction
GO: Gene Ontology
KEGG: Kyoto Encyclopaedia of Genes and Genomes
PDB: Protein Data Bank
VitB12: Vitamin B12
TCI: Tokyo Chemical Industry
HRP: Horseradish peroxidase
PRM: Parallel reaction monitoring
NCE: Normalised collision energies
PBS: Phosphate-buffered saline
DAB: Diaminobenzidine
RIPA: Radioimmunoprecipitation assay
BCA: Bicinchoninic acid
SDS-PAGE: Sodium dodecyl-sulphate polyacrylamide gel electrophoresis
PVDF: Polyvinylidene fluoride
BSA: Bovine serum albumin
TBST: Tris-buffered saline and Tween 20
cDNA: Complementary DNA
TNF-α: Tumour necrosis factor alpha
SD: Standard deviation
MF: Molecular function
BP: Biological process
CC: Cellular components
ROS: Reactive oxygen species
HIF: Hypoxia-inducible factor
BW: Body weight
FI: Food intake
